# Response to influenza immunization in patients with common variable immunodeficiency

**DOI:** 10.1186/1710-1492-7-S2-A28

**Published:** 2011-11-14

**Authors:** Wei Zhan, Fengyun Yue, Jun Liu, Jason K Lee, Jason Ho, Susan Moir, Mario A Ostrowski, Stephen D Betschel

**Affiliations:** 1 Department of Immunology, University of Toronto, Toronto, Ontario, M5S 1A8, Canada; 2 St. Michael’s Hospital, Toronto, Ontario, M5B 1W8, Canada; 3 National Institute of Allergy and Infectious Disease, National Institute of Health, Bethesda, Maryland, 20892, USA

## Background

Common variable immunodeficiency (CVID) is a heterogeneous primary immunodeficiency characterized by low serum antibody levels and recurrent infections. Cellular response to immunization in CVID has not been elucidated. In this study aimed to characterize influenza specific memory B-cell responses in patients with CVID and normal controls following influenza immunization.

## Methods

CVID and unaffected controls were immunized with the 2010 influenza vaccine. PBMCs were collected on the day of vaccination, and then week 8 and week 16 after vaccination. Memory B cell responses were determined by ELISPOT analysis.

## Results

Both the CVID and controls showed similar induction of flu-specific IgM-secreting memory B cells after vaccination. Before vaccination, CVID subjects had significantly lower frequencies of flu-specific IgG and IgA memory B cells. Half of the CVID subjects (4/8) showed an increase in flu-specific IgG-secreting memory B cells post vaccination, whereas the other half showed none. 8/8 controls showed increased flu-specific IgG-secreting memory B cells post-vaccination. None of the CVID subjects developed flu-specific IgA memory B cells post vaccination, compared to 5/8 normal subjects.

## Conclusions

A subgroup of CVID may be capable of making IgG memory responses to protein vaccination, although, the ability to maintain these responses needs to be studied with longer follow-up. Individuals with CVID however, demonstrated severe defects in IgA memory responses to vaccination, which may have clinical relevance in terms of protection against influenza. Further work is continuing to evaluate the influenza specific T-cell responses in this patient population.

